# Updated ACVIM consensus statement on equine herpesvirus‐1

**DOI:** 10.1111/jvim.17047

**Published:** 2024-03-18

**Authors:** David P. Lunn, Brandy A Burgess, David C. Dorman, Lutz S. Goehring, Peggy Gross, Klaus Osterrieder, Nicola Pusterla, Gisela Soboll Hussey

**Affiliations:** ^1^ School of Veterinary Science University of Liverpool Liverpool United Kingdom; ^2^ College of Veterinary Medicine University of Georgia Athens Georgia USA; ^3^ College of Veterinary Medicine North Carolina State University Raleigh North Carolina USA; ^4^ Maxwell H. Gluck Equine Research Center University of Kentucky, College of Agriculture, Food and Environment Lexington Kentucky USA; ^5^ Institut für Virologie Freie Universität Berlin Berlin Germany; ^6^ School of Veterinary Medicine University of California Davis California USA; ^7^ College of Veterinary Medicine Michigan State University, Veterinary Medical Center East Lansing Michigan USA

**Keywords:** abortion, diagnosis, equine, equine herpesvirus myeloencephalopathy, herpesvirus‐1, rhinopneumonitis, therapy, vaccination, viremia

## Abstract

Equine herpesvirus‐1 (EHV‐1) is a highly prevalent and frequently pathogenic infection of equids. The most serious clinical consequences of infection are abortion and equine herpesvirus myeloencephalopathy (EHM). The previous consensus statement was published in 2009 and considered pathogenesis, strain variation, epidemiology, diagnostic testing, vaccination, outbreak prevention and control, and treatment. A recent survey of American College of Veterinary Internal Medicine large animal diplomates identified the need for a revision to this original consensus statement. This updated consensus statement is underpinned by 4 systematic reviews that addressed key questions concerning vaccination, pharmaceutical treatment, pathogenesis, and diagnostic testing. Evidence for successful vaccination against, or effective treatment of EHV‐1 infection was limited, and improvements in experimental design and reporting of results are needed in future studies of this important disease. This consensus statement also updates the topics considered previously in 2009.

**Abbreviations**
ACVIM: American College of Veterinary Internal Medicine
CNS: Central Nervous System
CTL: Cytotoxic T‐lymphocyte
EHM: equine herpesvirus myeloencephalopathy
EHV‐1: equine herpesvirus‐1
GRADE: Grading of Recommendations, Assessment, Development, and Evaluation
LAIM: Large Animal Internal Medicine
MLV: Modified Live Vaccine
ORF: open reading frame
PICO: Population, Intervention, Comparator, and Outcome
PRISMA: Preferred Reporting Items for Systematic reviews and Meta‐Analyses
SNP: single‐nucleotide polymorphism
VN: Virus Neutralization

## 1 INTRODUCTION

Equine herpesvirus‐1 (EHV‐1, renamed: equid alphaherpesvirus‐1) infection is thought to be ubiquitous in equine populations throughout the world. Infection can occur in the first months of life^1^ and may result in the establishment of presumably lifelong latent infection. Primary infection, or reactivation of latent infection during periods of stress, causes clinical disease including rhinopneumonitis, epidemic abortion in late gestation, neonatal foal death, and equine herpesvirus myeloencephalopathy (EHM).^2^, ^3^, ^4^, ^5^ The ACVIM commissioned an EHV‐1 consensus statement in 2009,^6^ and there have been a series of international workshops focused on the virus, the most recent of which was in 2018.^7^ Although scientific investigation of the pathogenesis of diseases like EHM and their prevention continues, so do frequent outbreaks of disease in populations of horses across the world, including major outbreaks that have devastating effects on equine health and the equine industry.^8^, ^9^

The ongoing global effects of EHM led to the ACVIM Board of Regents charging the authors to revise the EHV‐1 consensus statement. The original EHV consensus statement was a literature review, structured around addressing a series of questions.^6^ The 9 questions addressed by the 2009 consensus statement and key messages are provided in Table 1. Some, but not all, of the answers to these questions that were provided in the 2009 consensus statement remain valid today. For example, factors to consider in controlling disease caused by EHV‐1 and best practices in response to an infection outbreak remain largely unchanged. In other cases, our scientific understanding of EHV‐1 infection in horses has improved resulting in updated messaging to equine practitioners. Our updates to these original 9 questions are included in this revised consensus statement.

**TABLE 1:** Questions and main conclusions in the 2009 EHV‐1 consensus statement.

Question	Main conclusions
How and why does equine herpesvirus‐1 (EHV‐1) infection target the pregnant uterus and CNS? Why do some horses but not others develop neurological disease?	Primary EHV‐1 infection occurs in epithelial cells of the upper respiratory tract (URT), with cell‐to‐cell spread leading to infection of URT lymph nodes within 24 to 48 hours. A mononuclear cell–associated viremia follows and can last up to 14 days. Nasal viral shedding typically ends by 10 to 14 days after infection. The viremia can infect endothelial cells of both the spinal cord resulting in equine herpesvirus myeloencephalopathy (EHM) or the pregnant uterus, resulting in abortion in the third trimester. The underlying pathogenesis is similar for both EHM and abortion. Incidence rates of EHM and abortion in infected horses are approximately 10% and ≥50%, respectively. The factors that lead to development of EHM are poorly understood, but in experimental infection studies, it occurs at much higher rates in old horses (18 years+).
What are the clinical implications of the DNA_pol_ single‐nucleotide polymorphism (SNP) (D_752_ versus N_752_)?	There is evidence of an association between a SNP in the DNA polymerase (DNApol) gene, leading to D752 and N752 biovars, which have been more often associated with EHM, and abortion, respectively. However, this association is not absolute and may be influenced by the relative prevalence of each strain in different populations. As a result, DNApol genotype is not relevant to the management and prevention of EHV‐1 disease outbreaks.
What does the most current data tell us about EHV‐1 epidemiology, and the prevalence of strain variants?	Reactivation from latency, with shedding and transmission to susceptible hosts are critical features of the epidemiology of EHV‐1 infection. Primary EHV‐1 infection occurs in the early months of life and may occur because of viral reactivation in latently infected mares. When horses are first infected, latency is established in both the lymphoreticular system and in the trigeminal ganglion. Clinicians should presume that most horses are latently infected with EHV‐1. Subclinical shedding of EHV‐1 is relatively infrequent.
What are the risk factors for horses for respiratory, abortigenic, or neurologic disease caused by EHV‐1?	Risk factors that influence the size and clinical presentation of EHV‐1 disease outbreaks include viral, host, and environmental factors. The presence of both EHV‐1 (eg, from an infected shedding horse) and susceptible horses is a prerequisite. Host factors also include breed, sex, reproductive status, and age. Younger horses (<2 years old) are at increased risk of developing respiratory disease, whereas older horses at increased risk of developing EHM. Abortion is largely restricted to the last trimester of pregnancy. Weaning, commingling, transportation, concurrent infections, and other stressors may increase infection rates. The role of immunity and EHM risk remains uncertain. Environmental factors include season with most EHM outbreaks occurring in late autumn, winter, and spring. Geographical region also appears to be associated with the development of EHM.
What kinds of viral detection tests should I select for diagnosis, prognosis, and screening of horses for EHV‐1 and its strains?	General recommendations for documenting an active EHV‐1 infection include the use of uncoagulated blood and nasal swab samples for quantitative real‐time PCR assays. These samples can be used for virus isolation of EHV‐1 when clinical signs and PCR results are suggestive of infection. Paired‐serum samples collected 15 to 21 days apart for serology—VN assay and ELISA for specific virus antigen, can provide presumptive evidence of EHV‐1 infection. In the absence of clinical signs consistent EHV‐1 infection, use of current diagnostic methods, including real‐time PCR, as a screening test is not recommended.
How and when should I use current commercially available vaccines to control EHV‐1 infection and disease?	Protection against EHV‐1 may require a combination of mucosal and systemic immune responses including both neutralizing antibody and CTL responses. Vaccination remains the optimal means to prevent infectious diseases; however, there is no evidence that vaccines prevent EHM, although there is some evidence for protection against abortion. Vaccination can be expected to reduce nasopharyngeal virus shedding during an outbreak and thereby limit the spread of infection.
What are the key factors to consider in controlling disease caused by EHV‐1?	Control measures include those designed to prevent or reduce the likelihood of outbreaks, and those designed to limit the spread of disease when an outbreak occurs. For example, measures used to prevent abortion or neurologic disease in pregnant mares include segregation of pregnant mares from other horses, isolation of all mares entering a stud facility for at least 3 weeks, subdivision of pregnant mares into small physically separated groups for the duration of gestation, maximize herd immunity through vaccination, and stress reduction. Similar measures can be applied to other horse populations.
What are the key things I need to know as I plan for, and respond to, an outbreak of clinical EHV‐1 infection?	The priorities for management of an outbreak of EHV‐1 are early diagnosis, prevention of further viral spread including disinfection of contaminated areas, isolation of infected horses and enhanced biosecurity to reduce the spread of infection, and management of clinical cases. Air‐borne, direct contact, and fomite transmission and contact with aborted fetuses, fetal membranes, and infected neonates are important modes of transmission. In the face of an EHV‐1 outbreak, vaccination can be used in horses at increased risk of exposure in the hope it may reduce spread of infectious virus. A period of 28 days after the occurrence of any new cases of EHV‐1 infection is recommended for the lifting of quarantine. Alternative strategies such as a 14‐day quarantine period followed by testing all horses by real‐time PCR analysis of nasal swabs for 2 to 4 consecutive days, along with twice daily monitoring of rectal temperatures has also been used. Horses that are dispersed to other stables should be quarantined on arrival and their health monitored. Virus in the environment is very unlikely to survive in an infectious form 21 days after depopulation of horses.
What therapeutic modalities are useful for treating EHM, beyond supportive and symptomatic care?	The treatment of horses with EHM involves empiric supportive care. The use of corticosteroids is reserved for EHM cases presenting in recumbency or with severe ataxia, in which the prognosis is guarded for survival. There is limited scientific rationale for the use of immunomodulators. Some antiviral drugs including the thymidine kinase inhibitor acyclovir have demonstrated in vitro efficacy against EHV‐1. However, evidence‐based studies of the value of antiviral drugs in the prevention and treatment of EHV‐1 infection are lacking. Acyclovir after a single oral administration to adult horses is associated with high variability in serum acyclovir‐time profiles and poor bioavailability. Bioavailability of valacyclovir is higher, although its impact on treatment outcome is unknown.

This revision uses a more rigorous evidence‐based approach that relies heavily on formal systematic review methodologies. This report describes our approaches and major findings from our literature reviews. We consider the implications for the findings of the first consensus statement, and we provide suggestions for future investigation and research.

### 1.1 Approach

The authors used an online survey tool to poll the Large Animal Internal Medicine (LAIM) ACVIM Diplomates to identify important challenges when managing EHV‐1 infection. Fifty‐one responses were received. Respondents were asked to evaluate the importance of the 9 questions addressed in the original EHV‐1 consensus statement,^6^ all of which were ranked as important by 82% to 96% of respondents. Half of the respondents also suggested additional high‐ and medium‐priority questions, and these responses were coded to identify themes. The authors of this consensus statement analyzed the responses and developed the following 4 high priority research questions that were judged to best address the challenges identified in the survey of LAIM ACVIM Diplomates:

1. Question 1: Does vaccination protect against EHV‐1 infection and disease?^10^
2. Question 2: Are pharmacologic treatments effective in managing EHV‐1 infection?^11^
3. Question 3: Does the degree of viremia correlate with the occurrence of abortion or EHM?^12^
4. Question 4: What is the best sampling strategy to detect EHV‐1 infection?^13^

### 1.2 Conduct of the reviews

Individual scoping and problem formulation steps were performed for each research question. Scoping considered stakeholder input from the survey and existing literature to determine the type of information available on the topic and to identify critical data gaps. Problem formulation helped refine the research questions and led to the development of Population, Intervention, Comparator, and Outcome (PICO) statements, inclusion and exclusion criteria, determined the type of evaluations to be performed, and identified the methods to be used for data management and extraction. Formal systematic reviews were performed to address Research Questions 1‐3. The remaining research question was addressed using a narrative review approach that included multiple systematic review elements. The systematic reviews followed the PRISMA (Preferred Reporting Items for Systematic Reviews and Meta‐Analyses) statement guidelines.^14^

Briefly, separate research protocols were developed for each review. A trained medical librarian (P. Gross) conducted literature searches of multiple databases for each review. Our reviews only considered peer‐reviewed publications in any language and without restriction to publication date. We did not include conference proceedings, technical reports, and other “gray” literature in our reviews. All citations were imported into Covidence systematic review software (Veritas Health Innovation, Melbourne, Australia) for screening by the research team. Screening was performed independently by 2 members of the research team. Citations were screened initially using review‐specific inclusion and exclusion criteria against material provided in the citation's title and abstract. Citations that were selected for the second tier of screening were evaluated using the full text of the study. Relevant data from citations that met an individual review's inclusion criteria were extracted. Studies included in our systematic reviews were evaluated for study quality using appropriate risk of bias assessment tools. Two systematic reviews that addressed the efficacy of vaccines and pharmacologic interventions were also evaluated using the Grading of Recommendations, Assessment, Development, and Evaluation (GRADE) approach to assess the strength of evidence for any finding.^15^ We adapted the GRADE approaches developed for animal studies by the National Institute of Environmental Health Sciences Office of Health Assessment and Translation and the National Academies of Sciences, Engineering, and Medicine.^16^, ^17^ Meta‐analyses were also performed to evaluate the efficacy of vaccines for different health outcomes.^10^ Additional information regarding the methods and findings of the 4 reviews are available in the individual manuscripts.^10^, ^11^, ^12^, ^13^

## 2 FINDINGS OF THE REVIEWS

Several global findings are worth noting. Our reviews relied heavily on experimental studies. These experiments varied by age of animals, reproductive status, breed of horses, strain of EHV‐1 used in challenges, types of interventions, outcomes of interest, timing of sample collection, among other factors resulting in considerable differences among study designs. This heterogeneity may contribute to the disparate results we often observed among individual studies evaluated in a review. We also found that reporting of the methodological features of individual studies was often incomplete, making evaluation of study quality within and across studies difficult. Researchers are encouraged to use ARRIVE guidelines (Animal Research: Reporting of In Vivo Experiments) to improve the reporting of research involving animals.^18^ Another challenge we faced in our reviews was inconsistent methods and approaches being used by different investigators. For example, case definitions for respiratory disease and EHM varied across studies. These differences may further contribute to the heterogeneity we observed and can also hamper the ability of investigators to synthesize the evidence using meta‐analytic approaches. Likewise, timing of when different outcome measures (eg, sampling for viremia, clinical evaluations) were assessed also varied among the reviewed studies. The reviewed experimental studies often had small sample sizes that led to some studies being underpowered. Moreover, incidence rates for EHM and abortion in many experimental studies were often low and further reduced our ability to detect treatment effects of these underpowered studies, which resulted in multiple studies reporting negative outcome data. It is clear that models of EHM and EHV‐1 abortion that will reliably induce the desired outcome need to be developed and used to more definitively answer questions considered in this consensus statement. Collectively, these finding often reduced our confidence in the available data, which is reflected in the current consensus statement. It has also led to our continuing advocacy for the use of certain interventions despite limited supporting data.

### 2.1 Research Question 1: Does vaccination protect against EHV‐1 infection and disease?

1. Evidence from the review: A total of 1018 unique studies were identified, of which 35 met the inclusion criteria.^10^ Experimental studies accounted for nearly 90% of the studies, with the remainder being observational studies. Eight vaccine subclasses were identified including commercial (modified live, inactivated, or a combination) and experimental (modified live, inactivated, deletion mutant MLV, DNA, recombinant) vaccines. Vaccine efficacy varied by both vaccine type and outcome. Several studies reported either no benefit or minimal vaccine efficacy for the primary outcomes of interest. Meta‐analyses revealed significant heterogeneity was present, and our overall confidence in the quality of the evidence for most outcomes was low to moderate. There is limited evidence that vaccination can reduce pyrexia and signs of respiratory disease and may reduce the levels and duration of nasal virus shedding. This evidence was strongest with modified live and deletion mutant vaccines. There is no evidence that vaccination fully prevents viremia and no evidence that it prevents the occurrence of EHM after EHV‐1 infection. There is limited evidence that killed vaccines may reduce the incidence of abortion. The findings of our systematic review are qualitatively similar to a recently published systematic review and meta‐analysis evaluating randomized controlled trials with experimental challenge of horses.^19^ This meta‐analysis showed that EHV‐1 vaccination did not result in significant improvements in clinical and virological outcomes.
2. Consensus statement: Our review indicates that commercial and experimental vaccines minimally reduce the incidence of clinical disease associated with EHV‐1 infection. Vaccination does not fully prevent horses from becoming EHV‐1 infected or prevent nasal shedding of virus after challenge infection. Importantly, our analysis is largely based on experimental studies that incompletely mimic conditions that occur naturally. Despite our systematic review finding that vaccine efficacy was often questionable, we support the vaccination of individual or groups of horses. Vaccination should be undertaken after a risk assessment of the likelihood of exposure to EHV‐1 infection and the consequences of infection. Vaccination may limit some signs of disease and the spread of infection, but it must be part of a comprehensive biosecurity program if EHV‐1 infection is to be prevented or limited. For at‐risk horses, vaccination is recommended as part of biosecurity program, and with an awareness of the limits of protection. Future research is critically needed to support development of safe and effective vaccines.

### 2.2 Research Question 2: Are pharmacologic treatments effective in managing EHV‐1 infection?

1. Evidence from the review: A total of 7009 unique studies were identified, of which 9 met the inclusion criteria.^11^ Two studies evaluated valacyclovir or small interfering RNAs, and single studies evaluated the use of a *Parapoxvirus ovis*–based immunomodulator, human alpha interferon, an herbal supplement, a cytosine analog, and heparin. Study designs included both randomized controlled studies and observational trials. Most studies reported either no benefit or minimal efficacy of the intervention tested. Our review indicates minimal or limited benefit either as a prophylactic or postexposure treatment for any of the studied interventions in the mitigation of EHV‐1–associated disease outcome.
2. Consensus statement: There is no evidence that pharmacologic treatments given after the onset of clinical signs of EHV‐1 infection prevent or affect the development or course of EHM. There is moderate evidence that valacyclovir given to horses in advance of EHV‐1 exposure can limit the development of EHM, and this strategy could be considered in an outbreak situation preexposure when early intervention is possible. Future research remains needed to evaluate the efficacy of pharmaceutical interventions under blinded, controlled, and randomized experimental conditions with sufficient sample size in the target population.

### 2.3 Research Question 3: Does the severity of viremia correlate with the occurrence of abortion or EHM?

1. Evidence from the review: A total of 189 unique studies were identified, of which 34 met the inclusion criteria.^12^ Thirty studies evaluated viremia and neurologic outcomes including 4 observational studies. Eight experimental studies examined viremia and abortion, which used the Ab4 and OH03 virus strains or mutant Ab4 derivatives. Several studies reported findings for both outcomes. Incidence rates for both EHM and abortion in experimental studies varied among the different studies as did the level of evidence. Viremia was generally detectable before the onset of either EHM or abortion. The results of our study support the hypothesis that viremia is regularly present before EHM or abortion occurs. However, no inferences could be made about the relationship between the occurrence of either signs of neurological disease or abortion and the magnitude or duration of viremia.
2. Consensus statement: Viremia is a prerequisite for the occurrence of both abortion and EHM. Therefore, prophylactic or therapeutic interventions that eliminate viremia are likely to also prevent abortions and EHM. There is limited current evidence that a reduction in the duration or magnitude of viremia will affect abortion or EHM outcomes.

### 2.4 Research Question 4: What is the best sampling technique to detect EHV‐1 infection?

1. Evidence from the review: 58 experimental and 19 observational studies met inclusion criteria.^13^ EHV‐1 detection frequency by quantitative PCR (qPCR) in nasal secretions and blood from naturally infected horses with fever and signs of respiratory disease were 15% and 9%, respectively; PCR detection rates in nasal secretions and blood from horses with suspected EHM were 94% and 70%, respectively. In contrast, when mares were tested after abortion, EHV‐1 was more frequently detected in blood samples when compared with nasal secretions. In experimental studies, the sensitivity of qPCR matched or exceeded that seen for virus isolation from either nasal secretions or blood. Detection of nasal shedding typically occurred within 2 days after EHV‐1 inoculation with a detection period of 3 to 7 days. Viremia lasted 2 to 4 days and was usually detected ≥1 days after positive identification of EHV‐1 in nasal secretions. Nasal shedding and viremia decreased over time and remained detectable in some horses for 2‐3 weeks after experimental infection. Under experimental conditions, blood and nasal secretions have similar sensitivity for the detection of EHV‐1 when horses are sampled on multiple consecutive days. In contrast, in observational studies detection of EHV‐1 in nasal secretions was consistently more successful.
2. Consensus statement: When horses are sampled on several consecutive days, such as is the case in experimental infections, both blood and nasal secretions display similar sensitivity for detection of EHV‐1 by qPCR. When horses are just sampled on 1 occasion, as often occurs in disease outbreaks, nasal secretion samples are more likely to detect EHV‐1 in horses with fever and signs of respiratory disease and in horses with suspected EHM. In contrast, in abortion outbreaks, blood samples are more successful for detecting EHV‐1. Experimental studies demonstrate that EHV‐1 can be detected in both blood and nasal secretions by qPCR for approximately 9 days after challenge infection, with viremia being initially detected 1 or more days after nasal shedding is first detected. When possible, it is advisable to test both nasal secretions and blood for the presence of EHV‐1 but when resources are limited or large numbers of horses have to be tested then only nasal secretions should be tested. The exception is in mares after abortion when blood samples are more sensitive than nasal secretions.

## 3 REVISION OF THE FIRST EHV‐1 CONSENSUS STATEMENT

The questions addressed by the first EHV‐1 consensus statement continue to be identified as important by an online survey of LAIM ACVIM Diplomates. A summary of the answers to these questions from the 2009 consensus statement^6^ can be found in Table 1. The sections below seek to identify new peer‐reviewed papers published since the first statement that inform the answers to the questions.

### 3.1 Pathogenesis: How and why does EHV‐1 infection target the pregnant uterus and CNS? Why do some horses but not others develop neurological disease?

After primary infection of the respiratory epithelium, EHV‐1 infection is established in local respiratory lymph nodes within 24 to 28 hours. A cell‐associated viremia is then established with EHV‐1 present in CD8 and CD4 lymphocytes, B lymphocytes, and monocytes.^20^ This viremia transports the virus to the vascular endothelium of secondary sites of infection, which are typically immune privileged tissues. Infection of the pregnant uterus and the CNS resulting in third trimester abortion and EHM are the most important secondary sites, but the vasculature of eye and the testis are also affected.^21^, ^22^

The pathogenic mechanism underlying CNS endothelial infection remains ill defined. Viral factors have been reported to affect pathogenesis, including the extensively studied D752/N752 single‐nucleotide polymorphism (SNP) in the DNA polymerase (DNA Pol) described in the next section. Additional studies of mutations in the open reading frame (ORF) 30 gene and their association with EHM have been published,^23^, ^24^ but it has not been clearly demonstrated that any EHV‐1 genotype is associated with increased pathogenicity. Other viral proteins have been implicated in the neuropathogenesis of EHV‐1 including glycoprotein D in an equine model,^25^ and glycoprotein B, the protein kinase US3, and the ORF1/2 genes in in vitro studies, but their association with disease progression or severity is also unclear.^26^, ^27^ A review of viral factors putatively involved in neuropathogenesis was recently published.^28^

### 3.2 Neuropathogenic strains: What are the clinical implications of the DNA Pol variants (D_752_ versus N_752_ )?

In 2006, a comprehensive study of EHV‐1 genotypes from several continents proposed that a point mutation in the DNA Pol, the virus enzyme responsible for replicating the virus' genetic material, might be a bona fide virulence marker.^29^ A single nonsynonymous nucleotide exchange (adenine at position 2254 to guanine) resulting in an amino acid change at position 752 (asparagine to its acidic cousin aspartic acid) of the 1220 amino acid protein, appeared to be more frequently associated with EHV‐1 infections that resulted in EHM. A biological explanation was provided inasmuch as D752 variants replicated more robustly in some target cells, specifically in peripheral blood mononuclear cells.^29^, ^30^, ^31^ However, several subsequent studies of EHV‐1 isolates challenge the tenet that the D752 EHV‐1 biovar is more frequently associated with EHM cases, at least in certain geographic locations.^24^, ^32^, ^33^, ^34^ For example, a recent large outbreak of EHM that originated at an international jumping event in Valencia, Spain, and then spread extensively was caused by a representative of the N752 biovar.^9^, ^35^, ^36^ Also of note, investigations of EHV‐1 isolates from other recent outbreaks revealed a third SNP at position 2254 (cytosine) of DNA Pol, which results in an H752, although the effects, if any, on replicative ability or pathogenic potential remains uncertain.^24^, ^37^, ^38^

Currently, it is not clear that any specific EHV‐1 strain is more likely to cause neurological disease or that any of the 3 DNA Pol variants (D752, N752, or H752) differ markedly in their ability to cause EHM outbreaks in the field. To better understand the role of EHV‐1 strains in naturally occurring disease, we need to know the prevalence of strains in different equine populations to determine whether a disease association with a strain is a result of strain pathogenicity or more simply of a high prevalence of a strain in a population.

### 3.3 Epidemiology: What does the most current data tell us about EHV‐1 epidemiology and the prevalence of strain variants?

The epidemiology of EHV‐1 is believed to depend on latency and reactivation, neither of which are well understood. Primary EHV‐1 infections occur early in life, but it is unclear what percentage of infections result in the establishment of latency, that is, virus persistence in horses or how long this persistent state is maintained. Factors leading to reactivation in the field are unknown. When latent EHV‐1 infection is reactivated by immunosuppression with corticosteroids under experimental conditions, subsequent spread to other susceptible horses did not occur.^39^ Latency becomes established in lymphoreticular tissues and trigeminal ganglia.^6^ The percentage of horses harboring latent infections appears highly variable and may depend on the geographic region, the population tested, and the technology and criteria used to detect this quiescent state of infection. Although many authorities suggest the prevalence of latent EHV‐1 infection in adult horses to be in excess of 60%,^4^, ^5^, ^6^ more recent publications have estimated that the percentage of latently infected horses is much smaller and ranges from 15% to 27%, with latent EHV‐1 detected in the trigeminal ganglia more commonly than in submandibular and bronchial lymph nodes.^40^, ^41^ The rate of subclinical EHV‐1 shedding in horses is low and depends on the time of the year as well as the age and use of the population of horses tested.^42^, ^43^, ^44^, ^45^, ^46^, ^47^, ^48^ Large outbreaks of EHM remain infrequent, although their effects on the equine industry in Europe and the USA continues to raise concern, and many questions surrounding risk factors, prevention, and management remain unanswered.^8^, ^49^

### 3.4 Risk factors for disease: What are the risk factors for horses for respiratory, abortigenic, or neurologic disease caused by EHV‐1

The risk factors identified in the 2009 EHV‐1 consensus statement included viral, host and environmental factors,^6^ and the known factors are largely unchanged. One study by Klouth et al^50^ again identified increasing age, fever, and female sex as risk factors for occurrence of EHM and indicated that Welsh, and Shetland ponies, were at a lower risk. Large multiday gatherings of horses were associated with the 2 notable very large EHM outbreaks in 2011 and 2022.^8^, ^49^ An experimental study identified a series of immunoregulatory changes in horses that developed EHM.^51^ A review of host factor contribution to neuropathogenesis was published in 2022.^52^

### 3.5 Diagnostic testing: What kinds of viral detection tests should I select for diagnosis, prognosis, and screening of horses for EHV‐1 and its strains?

Real‐time qPCR has supplanted conventional PCR and virus culture for the detection of EHV‐1 in both respiratory secretions and uncoagulated blood as a diagnostic tool because of its high analytical sensitivity and specificity. Nevertheless, virus isolation should ideally still be attempted at reference laboratories for detailed characterization of the virus isolate. This consensus statement recommends testing of both nasal secretions and blood samples for the presence of EHV‐1 with some caveats as stated in our findings above.

Consequent to the identification of the DNApol SNP (D752/N752/H752), commercially available tests have been developed that can distinguish these 3 biovars^24^, ^53^; however, the interpretation of the results in terms of disease association needs caution as discussed under “Neuropathogenic Strains” above. To further reduce the turn‐around‐time of sample testing, various stall‐side molecular EHV‐1 testing platforms have been described,^54^, ^55^ and new technologies may be applicable to these platforms.^56^ Although rapid testing is advantageous in acute case management, it is important that new point‐of‐care EHV‐1 tests are properly validated and show acceptable agreement with gold standard tests, including qPCR and virus isolation techniques.

### 3.6 Vaccination: How, and when should I use current commercially available vaccines to control EHV‐1 infection and disease?

The recommendations of the original consensus statement remain relevant.^6^ Vaccination against EHV‐1 infection is likely to be effective in preventing the major pathological sequelae when it can prevent the occurrence of viremia, as supported by our consensus opinion on Research Question 3 above and our recent systematic review.^12^ The prevention of viremia may be a reasonable surrogate for abortion and EHM challenge models in horses and offer a more ethically acceptable experimental endpoint.

Our current consensus statement on the efficacy of EHV‐1 vaccination is presented above, and this is supported by 2 recently published systematic reviews of EHV‐1 vaccine efficacy.^10^, ^19^ Although our overall confidence in the quality of the evidence was low to moderate, we continue to recommend vaccination as part of biosecurity program, with an awareness of the limits of protection, and after a risk assessment of the likelihood of exposure to EHV‐1 infection and the consequences of infection.

### 3.7 Disease control and prevention: What are the key factors to consider in controlling disease caused by EHV‐1?

The fundamental principles described in the original consensus statement remain unchanged (Table 1).^6^

### 3.8 Outbreak response: What are the key things I need to know as I plan for, and respond to, an outbreak of clinical EHV‐1 infection?

The fundamental principles of outbreak response described in the original consensus statement remain unchanged (Table 1),^6^ and the AAEP General Biosecurity Guidelines are a good resource when confronted by the risk or diagnosis of EHV‐1.^57^ Strategies aimed at preparing for and responding to an EHV‐1 outbreak continue to include early diagnosis, prevention of further spread, and management of clinical cases. The diagnostic techniques best suited to detecting EHV‐1 infection are described above, and the clinician needs to identify a laboratory that can provide testing in advance of having to use their services.

Our understanding of the duration and intensity of EHV‐1 shedding by infected horses has changed consequent to experiences gained during major outbreaks of EHM at veterinary hospitals and large boarding facilities.^8^, ^58^, ^59^, ^60^ Infected horses in EHM outbreaks can shed infectious amounts of EHV‐1 for many days beyond the onset of clinical disease. Our ability to prevent this spread among horses in the same building, even with extensive barrier precautions, is limited. It is therefore important to house suspected and confirmed EHV‐1 cases in isolation facilities whenever practicable and to stop horse movement and practice stringent biosecurity for all in contact animals pending the outcome of testing or completion of quarantine.

The risks of environmental spread of EHV‐1 have recently been reviewed.^61^ Potentially infectious EHV‐1 persists in the environment for at least 48 hours^62^ and for up to 14 days in water.^63^ Although virus in the environment is unlikely to survive in an infectious form 21 days after removal of horses, appropriate disinfection and decontamination of facilities will be an important component of outbreak control in most circumstances.

### 3.9 Treatment: What therapeutic modalities are useful for treating EHM, beyond supportive and symptomatic care?

Our current consensus statement on the value of pharmacologic treatments for EHV‐1 infection is presented above and is supported by our recently published systematic review.^11^

## 4 CONCLUSIONS

Equine herpesvirus‐1 infection remains an important equine pathogen throughout most of the world, and an active area of investigation. Although important progress has been made in several areas, our ability to vaccinate against, or treat the sequelae of infection with EHV‐1 remains limited.

We encourage investigators to consider our reviews when designing future studies. As noted earlier, our reviews often found inconsistent results, moderate to high risk of bias and heterogeneity among the studies we reviewed. Robust studies with adequate statistical power remain of utmost importance to reliably determine whether vaccination or therapeutic interventions are effective in the management of EHV‐1 infection in horses. More thorough reporting of study methods and results using available reporting guidelines^18^ is also desirable. Another lesson learned from our reviews is the need for additional sampling across time to better characterize disease outcomes and progression. Publication of individual animal data as supplemental files will also support future systematic reviews and analyses.

## CONFLICT OF INTEREST DECLARATION

Authors declare no conflict of interest.

## OFF‐LABEL ANTIMICROBIAL DECLARATION

Authors declare no off‐label use of antimicrobials.

## INSTITUTIONAL ANIMAL CARE AND USE COMMITTEE (IACUC) OR OTHER APPROVAL DECLARATION

Authors declare no IACUC or other approval was needed.

## HUMAN ETHICS APPROVAL DECLARATION

Authors declare human ethics approval was not needed for this study.

## References

[1] Foote CE , Love DN , Gilkerson JR , Whalley JM . Detection of EHV‐1 and EHV‐4 DNA in unweaned thoroughbred foals from vaccinated mares on a large stud farm. Equine Vet J. 2004;36:341‐345. 15163042 10.2746/0425164044890634

[2] Kydd JH , Townsend HG , Hannant D . The equine immune response to equine herpesvirus‐1: the virus and its vaccines. Vet Immunol Immunopathol. 2006;111:15‐30. 16476492 10.1016/j.vetimm.2006.01.005

[3] Slater JD , Lunn DP , Horohov DW , et al. Report of the equine herpesvirus‐1 Havermeyer workshop, San Gimignano, Tuscany, June 2004. Vet Immunol Immunopathol. 2006;111:3‐13. 16542736 10.1016/j.vetimm.2006.01.004

[4] Allen GP , Kydd JH , Slater JD , Smith KC . Equid herpesvirus 1 and equid herpesvirus 4 infections. In: Coetzer JAW , Tustin RC , eds. Infectious Diseases of Livestock. 1st ed. Oxford University Press; 2004:829‐859.

[5] Slater JD . Equine herpesviruses. In: Sellon DC , Long MT , eds. Equine Infectious Diseases. Saunders Elsevier; 2007:134‐152.

[6] Lunn DP , Davis‐Poynter N , Flaminio MJBF , et al. Equine herpesvirus‐1 consensus statement. J Vet Intern Med. 2009;23:450‐461. 19645832 10.1111/j.1939-1676.2009.0304.x

[7] Kydd JH , Lunn DP , Osterrieder K . Report of the fourth international Havemeyer workshop on equid herpesviruses (EHV) EHV‐1, EHV‐2 and EHV‐5. Equine Vet J. 2019;51:565‐568. 31373057 10.1111/evj.13141

[8] Traub‐Dargatz JL , Pelzel‐McCluskey AM , Creekmore LH , et al. Case‐control study of a multistate equine herpesvirus myeloencephalopathy outbreak. J Vet Intern Med. 2013;27:339‐346. 23398291 10.1111/jvim.12051

[9] European Food Safety Authority (EFSA) , Carvelli A , Nielsen SS , Paillot R , Broglia A , Kohnle L . Clinical impact, diagnosis and control of equine Herpesvirus‐1 infection in Europe. EFSA J. 2022;20:e07230. 35414834 10.2903/j.efsa.2022.7230 PMC8985062

[10] Osterrieder K , Dorman DC , Burgess BA , et al. Vaccination for the prevention of equine herpesvirus‐1 disease in domesticated horses: a systematic review and meta‐analysis. J Vet Intern Med. 2024;38(3):1858‐1871. doi:10.1111/jvim.16895 37930113 PMC11099739

[11] Goehring L , Dorman DC , Osterrieder K , et al. Pharmacologic interventions for the treatment of equine herpesvirus‐1 in domesticated horses: a systematic review. J Vet Intern Med. 2024;38(3):1892‐1905. doi:10.1111/jvim.17016 38380685 PMC11099759

[12] Soboll‐Hussey G , Dorman DC , Burgess BA , et al. Relationship between equine herpesvirus‐1 viremia and abortion or equine herpesvirus myeloencephalopathy in domesticated horses: a systematic review. J Vet Intern Med. 2024;38(3):1872‐1891. doi:10.1111/jvim.16948 38069576 PMC11099755

[13] Pusterla N , Dorman DC , Burgess BA , et al. Viremia and nasal shedding for the diagnosis of equine herpesvirus‐1 infection in domesticated horses. J Vet Intern Med. 2024;38(3):1765‐1791. doi:10.1111/jvim.16958 38069548 PMC11099742

[14] Page MJ , McKenzie JE , Bossuyt PM , et al. The PRISMA 2020 statement: an updated guideline for reporting systematic reviews. BMJ. 2021;372:n71. 33782057 10.1136/bmj.n71 PMC8005924

[15] Brożek JL , Akl EA , Alonso‐Coello P , et al. Grading quality of evidence and strength of recommendations in clinical practice guidelines. Part 1 of 3. An overview of the GRADE approach and grading quality of evidence about interventions. Allergy. 2009;64:669‐677. 19210357 10.1111/j.1398-9995.2009.01973.x

[16] Office of Health Assessment and Translation DotNTP, National Institute of Environmental Health Sciences . Handbook for Conducting a Literature‐Base Health Assessment Using OHAT Approach for Systematic Review and Evidence Integration, ed. 2019. https://ntp.niehs.nih.gov/sites/default/files/ntp/ohat/pubs/handbookmarch2019_508.pdf, accessed March 2, 2024.

[17] National Academies of Sciences, Engineering, and Medicine . Application of Systematic Review Methods in an Overall Strategy for Evaluating Low‐Dose Toxicity from Endocrine Active Chemicals. The National Academies Press; 2017. 28896009

[18] Percie du Sert N , Hurst V , Ahluwalia A , et al. The ARRIVE guidelines 2.0: updated guidelines for reporting animal research. PLoS Biol. 2020;18:e3000410. 32663219 10.1371/journal.pbio.3000410 PMC7360023

[19] Marenzoni ML , Passamonti F , Cappelli K , et al. Clinical, serological and molecular investigations of EHV‐1 and EHV‐4 in 15 unweaned thoroughbred foals. Vet Rec. 2008;162:337‐341. 18344498 10.1136/vr.162.11.337

[20] Wilsterman S , Soboll‐Hussey G , Lunn DP , et al. Equine herpesvirus‐1 infected peripheral blood mononuclear cell subpopulations during viremia. Vet Microbiol. 2011;149:40‐47. 21093993 10.1016/j.vetmic.2010.10.004

[21] Holz CL , Sledge DG , Kiupel M , Nelli RK , Goehring LS , Soboll Hussey G . Histopathologic findings following experimental equine herpesvirus 1 infection of horses. Front Vet Sci. 2019;6:59. 30886853 10.3389/fvets.2019.00059 PMC6409500

[22] Hussey GS , Goehring LS , Lunn DP , et al. Experimental infection with equine herpesvirus type 1 (EHV‐1) induces chorioretinal lesions. Vet Res. 2013;44:118. 24308772 10.1186/1297-9716-44-118 PMC4028784

[23] Pronost S , Léon A , Legrand L , Miszczak F , Freymuth F , Fortier G . Neuropathogenic and non‐neuropathogenic variants of equine herpesvirus 1 in France. Vet Microbiol. 2010;145:329‐333. 20427133 10.1016/j.vetmic.2010.03.031

[24] Pusterla N , Barnum S , Lawton K , Wademan C , Corbin R , Hodzic E . Investigation of the EHV‐1 genotype (N(752), D(752), and H(752)) in swabs collected from equids with respiratory and neurological disease and abortion from the United States (2019‐2022). J Equine Vet. 2023;123:104244. 10.1016/j.jevs.2023.104244 36773852

[25] Holz CL , Nelli RK , Wilson ME , et al. Viral genes and cellular markers associated with neurological complications during herpesvirus infections. J Gen Virol. 2017;98:1439‐1454. 28631601 10.1099/jgv.0.000773

[26] Spiesschaert B , Goldenbogen B , Taferner S , et al. Role of gB and pUS3 in EHV‐1 transfer between PBMC and endothelial cells: a dynamic in vitro model. J Virol. 2015;89:11899‐11908. 26378176 10.1128/JVI.01809-15 PMC4645325

[27] Pavulraj S , Kamel M , Stephanowitz H , et al. Equine herpesvirus type 1 modulates cytokine and chemokine profiles of mononuclear cells for efficient dissemination to target organs. Viruses. 2020;12:12. 10.3390/v12090999 PMC7551999 32911663

[28] Laval K , Poelaert KCK , van Cleemput J , et al. The pathogenesis and immune evasive mechanisms of equine herpesvirus type 1. Front Microbiol. 2021;12:662686. 33746936 10.3389/fmicb.2021.662686 PMC7970122

[29] Nugent J , Birch‐Machin I , Smith KC , et al. Analysis of equid herpesvirus 1 strain variation reveals a point mutation of the DNA polymerase strongly associated with neuropathogenic versus nonneuropathogenic disease outbreaks. J Virol. 2006;80:4047‐4060. 16571821 10.1128/JVI.80.8.4047-4060.2006 PMC1440451

[30] Goodman LB , Loregian A , Perkins GA , et al. A point mutation in a herpesvirus polymerase determines neuropathogenicity. PLoS Pathog. 2007;3:e160. 17997600 10.1371/journal.ppat.0030160 PMC2065875

[31] van de Walle GR , Goupil R , Wishon C , Damiani A , Perkins GA , Osterrieder N . A single‐nucleotide polymorphism in a herpesvirus DNA polymerase is sufficient to cause lethal neurological disease. J Infect Dis. 2009;200:20‐25. 19456260 10.1086/599316

[32] Vissani MA , Becerra ML , Olguín Perglione C , Miño S , Barrandeguy M . Neuropathogenic and non‐neuropathogenic genotypes of equid herpesvirus type 1 in Argentina. Vet Microbiol. 2009;139:361‐364. 19589651 10.1016/j.vetmic.2009.06.025

[33] Fritsche AK , Borchers K . Detection of neuropathogenic strains of equid herpesvirus 1 (EHV‐1) associated with abortions in Germany. Vet Microbiol. 2010;147:176‐180. 20619972 10.1016/j.vetmic.2010.06.014

[34] Walter J , Seeh C , Fey K , Bleul U , Osterrieder N . Clinical observations and management of a severe equine herpesvirus type 1 outbreak with abortion and encephalomyelitis. Acta Vet Scand. 2013;55:19. 23497661 10.1186/1751-0147-55-19 PMC3630004

[35] Kubacki J , Lechmann J , Fraefel C , Bachofen C . Genome sequence of equid Alphaherpesvirus 1 (EHV‐1) from a nasal swab of a Swiss horse associated with a major EHV‐1 outbreak following a show jumping event in Valencia, Spain. Microbiol Resour Announc. 2021;10:e0073221. 34435856 10.1128/MRA.00732-21 PMC8388538

[36] Vereecke N , Carnet F , Pronost S , Vanschandevijl K , Theuns S , Nauwynck H . Genome sequences of equine herpesvirus 1 strains from a European outbreak of neurological disorders linked to a horse gathering in Valencia, Spain, in 2021. Microbiol Resour Announc. 2021;10:e00333‐21. 34016681 10.1128/MRA.00333-21 PMC8188346

[37] Sutton G , Normand C , Carnet F , et al. Equine herpesvirus 1 variant and new marker for epidemiologic surveillance, Europe, 2021. Emerg Infect Dis. 2021;27:2738‐2739. 34546162 10.3201/eid2710.210704 PMC8462333

[38] Hu Y , Jia Q , Liu J , et al. Molecular characteristics and pathogenicity of an equid alphaherpesvirus 1 strain isolated in China. Virus Genes. 2022;58:284‐293. 35567668 10.1007/s11262-022-01910-y

[39] Pusterla N , Hussey SB , Mapes S , et al. Molecular investigation of the viral kinetics of equine herpesvirus‐1 in blood and nasal secretions of horses after corticosteroid‐induced recrudescence of latent infection. J Vet Intern Med. 2010;24:1153‐1157. 20584139 10.1111/j.1939-1676.2010.0554.x

[40] Pusterla N , Mapes S , Wilson WD . Prevalence of equine herpesvirus type 1 in trigeminal ganglia and submandibular lymph nodes of equids examined postmortem. Vet Rec. 2010;167:376‐378. 20817899 10.1136/vr.c3748

[41] Pusterla N , Mapes S , David Wilson W . Prevalence of latent alpha‐herpesviruses in thoroughbred racing horses. Vet J. 2012;193:579‐582. 22405721 10.1016/j.tvjl.2012.01.030

[42] Pusterla N , Barnum S , Young A , et al. Molecular monitoring of EHV‐1 in silently infected performance horses through nasal and environmental sample testing. Pathogens. 2022;11:720. 35889966 10.3390/pathogens11070720 PMC9317758

[43] Pusterla N , Sandler‐Burtness E , Barnum S , et al. Frequency of detection of respiratory pathogens in nasal secretions from healthy sport horses attending a spring show in California. J Equine Vet. 2022;117:104089. 10.1016/j.jevs.2022.104089 35908600

[44] Stout AE , Hofmar‐Glennon HG , André NM , et al. Infectious disease surveillance of apparently healthy horses at a multi‐day show using a novel nanoscale real‐time PCR panel. J Vet Diagn Invest. 2021;33:80‐86. 33179576 10.1177/1040638720972096 PMC7758683

[45] Couetil L , Ivester K , Barnum S , Pusterla N . Equine respiratory viruses, airway inflammation and performance in thoroughbred racehorses. Vet Microbiol. 2021;257:109070. 33865081 10.1016/j.vetmic.2021.109070

[46] Smith FL , Watson JL , Spier SJ , et al. Frequency of shedding of respiratory pathogens in horses recently imported to the United States. J Vet Intern Med. 2018;32:1436‐1441. 29761571 10.1111/jvim.15145 PMC6060314

[47] Stasiak K , Dunowska M , Rola J . Prevalence and sequence analysis of equid herpesviruses from the respiratory tract of polish horses. Virol J. 2018;15:106. 29996858 10.1186/s12985-018-1018-3 PMC6042439

[48] Carr E , Schott H , Pusterla N . Absence of equid herpesvirus‐1 reactivation and viremia in hospitalized critically ill horses. J Vet Intern Med. 2011;25:1190‐1193. 21848945 10.1111/j.1939-1676.2011.0775.x

[49] Couroucé A , Normand C , Tessier C , et al. Equine Herpesvirus‐1 outbreak during a show‐jumping competition: a clinical and epidemiological study. J Equine Vet. 2023;128:104869. 10.1016/j.jevs.2023.104869 37339699

[50] Klouth E , Zablotski Y , Petersen JL , et al. Epidemiological aspects of equid herpesvirus‐associated myeloencephalopathy (EHM) outbreaks. Viruses. 2022;14:14. 10.3390/v14112576 PMC9695031 36423188

[51] Zarski LM , Giessler KS , Jacob SI , et al. Identification of host factors associated with the development of equine herpesvirus Myeloencephalopathy by transcriptomic analysis of peripheral blood mononuclear cells from horses. Viruses. 2021;13:13. 10.3390/v13030356 PMC7995974 33668216

[52] Hussey GS , Giessler KS . Contribution of the immune response to the pathogenesis of equine herpesvirus‐1 (EHV‐1): are there immune correlates that predict increased risk or protection from EHV‐1 myeloencephalopathy? Vet J. 2022;282:105827. 35405348 10.1016/j.tvjl.2022.105827

[53] Sutton G , Thieulent C , Fortier C , et al. Identification of a new equid herpesvirus 1 DNA polymerase (ORF30) genotype with the isolation of a C‐2254/H‐752 strain in French horses showing no major impact on the strain behaviour. Viruses. 2020;12:1160. 33066315 10.3390/v12101160 PMC7650556

[54] Chen W , Yu H , Sun F , et al. Mobile platform for multiplexed detection and differentiation of disease‐specific nucleic acid sequences, using microfluidic loop‐mediated isothermal amplification and smartphone detection. Anal Chem. 2017;89:11219‐11226. 28819973 10.1021/acs.analchem.7b02478

[55] Sun F , Ganguli A , Nguyen J , et al. Smartphone‐based multiplex 30‐minute nucleic acid test of live virus from nasal swab extract. Lab Chip. 2020;20:1621‐1627. 32334422 10.1039/d0lc00304b

[56] Ghoniem SM , ElZorkany HE , Hagag NM , el‐Deeb AH , Shahein MA , Hussein HA . Development of multiplex gold nanoparticles biosensors for ultrasensitive detection and genotyping of equine herpes viruses. Sci Rep. 2023;13:15140. 37704638 10.1038/s41598-023-41918-4 PMC10500010

[57] Jones B , Flynn K , Pelzel‐McCluskey AM , Traub‐Dargatz J , White N . AAEP general biosecurity guidelines. 2022. https://aaep.org/document/general-biosecurity-guidelines, accessed March 2, 2024.

[58] Goehring LS , Landolt GA , Morley PS . Detection and management of an outbreak of equine herpesvirus type 1 infection and associated neurological disease in a veterinary teaching hospital. J Vet Intern Med. 2010;24:1176‐1183. 20584137 10.1111/j.1939-1676.2010.0558.x

[59] Burgess BA , Tokateloff N , Manning S , et al. Nasal shedding of equine herpesvirus‐1 from horses in an outbreak of equine herpes myeloencephalopathy in Western Canada. J Vet Intern Med. 2012;26:384‐392. 22332764 10.1111/j.1939-1676.2012.00885.x

[60] Vandenberghe E , Boshuizen B , Delesalle CJG , et al. New insights into the management of an EHV‐1 (equine hospital) outbreak. Viruses. 2021;13:13. 10.3390/v13081429 PMC8402800 34452295

[61] Dayaram A , Seeber PA , Greenwood AD . Environmental detection and potential transmission of equine herpesviruses. Pathogens. 2021;10:423. 33916280 10.3390/pathogens10040423 PMC8066653

[62] Saklou NT , Burgess BA , Ashton LV , Morley PS , Goehring LS . Environmental persistence of equid herpesvirus type‐1. Equine Vet J. 2021;53:349‐355. 32557765 10.1111/evj.13313

[63] Dayaram A , Franz M , Schattschneider A , et al. Long term stability and infectivity of herpesviruses in water. Sci Rep. 2017;7:46559. 28429732 10.1038/srep46559 PMC5399353

